# Endothelial system dysregulation underlies keloid development and therapeutic targeting

**DOI:** 10.3389/fcell.2026.1919403

**Published:** 2026-09-10

**Authors:** Yongxin Yu, Juexian Zhu, Long Ma, Xiangyun Zhang, Hong Yan

**Affiliations:** 1 Department of Plastic and Aesthetic Repair and Reconstruction, West China Second University Hospital, Sichuan University, Chengdu, China; 2 Key Laboratory of Birth Defects and Related Diseases of Women and Children (Sichuan University), Ministry of Education, Chengdu, China

**Keywords:** endothelial system dysfunction, endothelial-to-mesenchymal transition, keloid, therapeutic strategies, pathological remodeling of blood and lymphatic vessels

## Abstract

Cutaneous keloids are pathological scars resulting from aberrant wound healing, characterized by infiltrative growth beyond the original wound margins and a failure to regress spontaneously. Although traditionally attributed to excessive extracellular matrix deposition and fibroblast proliferation, emerging evidence underscores the pivotal role of vascular dysregulation and endothelial dysfunction in keloid pathogenesis. This review systematically examines the endothelial cell system—comprising vascular endothelial cells (VECs) and lymphatic endothelial cells (LECs)—within the keloid microenvironment, focusing on how hypoxia, chronic inflammation, mechanical forces, sex hormones, the renin–angiotensin system, and neuropeptides converge to drive structural and functional endothelial abnormalities. Special emphasis is placed on endothelial-to-mesenchymal transition (EndMT) as a critical pathway linking vascular pathology to fibrotic progression. Additionally, we comprehensively summarize current and emerging therapeutic strategies targeting the endothelium, including anti-angiogenic agents, HIF-1α inhibition, EndMT blockade, mechanical offloading, hormonal modulation, renin–angiotensin system (RAS) inhibition, and laser therapy. By providing an integrated overview of the endothelial mechanisms underlying keloid formation and persistence, this review aims to illuminate novel targets for therapeutic intervention and to inform the development of more effective clinical management strategies.

## 1 Introduction

Cutaneous keloids are pathological scars resulting from aberrant wound healing, characterized by chronic inflammation and excessive deposition of extracellular matrix (ECM) (Kim and Kim, 2024). Keloids extend beyond the original wound margins, infiltrating surrounding normal skin; they grow for years or decades without spontaneous regression, and are therefore considered benign tumors (Tan et al., 2019; Ogawa and Akaishi, 2016). Keloids commonly occur in areas such as the sternal region, shoulder girdle, back, earlobes, and neck, resulting in cosmetic disfigurement. Severe cases may also be accompanied by symptoms of pain and pruritus, and can even lead to functional impairment of the joints (Zhang et al., 2024; Butzelaar et al., 2017),and profoundly affect patients’ physical and mental health. Although various treatment modalities—such as surgical excision, corticosteroid injection, laser therapy, and radiotherapy—are available, their efficacy is often unsatisfactory and recurrence rates remain high (Wang et al., 2025a; Huang et al., 2019). Previous research has predominantly focused on dermal fibroblasts as the primary cellular mediator of keloid formation (Wen et al., 2025). However, with deeper understanding, keloids are now recognized as products of a complex, multi-cellular and multi-factorial network (Zhang et al., 2023). Within this network, the endothelial cell system—comprising vascular endothelial cells (VECs) and lymphatic endothelial cells (LECs)—plays an increasingly central role in the keloid microenvironment. Notably, while most existing studies have concentrated on VECs and their crosstalk with fibroblasts (Cheng et al., 2024; Li et al., 2025), and the role of LECs has received growing attention, the potential synergistic interplay between VECs and LECs during keloid pathogenesis remains virtually uncharted in the literature. This prevailing single-cell-type perspective overlooks the collective pathogenic force exerted by the endothelial system as an integrated regulatory unit, wherein the complementary, open-channel networks of VECs and LECs coordinately shape the microenvironment (Morfoisse and Noel, 2019). Therefore, this review aims to transcend the limitations of isolated cell-type analyses by systematically examining the combined role of the VEC-LEC endothelial system in keloid pathogenesis.

In the keloid microenvironment, VECs exhibit structural remodeling and functional abnormalities. Disruption of intercellular junctions increases vascular permeability, establishing the pathological basis for tissue edema and inflammatory cell infiltration (Wen et al., 2025). Although there is currently no direct evidence linking impaired lymphatic drainage to the development of keloids, studies have shown that lymphatic dysfunction leads to the accumulation of interstitial fluid and persistent tissue edema; subsequently, the accumulation of proteins and inflammatory mediators stimulates fibroblasts to produce collagen, ultimately resulting in fibrosis (Granoski et al., 2024). Therefore, it can be inferred that the integrity of lymphatic drainage function is crucial for limiting the inflammatory response and maintaining the stability of the local microenvironment. Under the influence of multiple factors—including a hypoxic microenvironment, persistent inflammation (Sone et al., 2025), endothelial-to-mesenchymal transition (EndMT) (Guo et al., 2023), mechanical forces (Zhang et al., 2024), hormones (Hedayatyanfard et al., 2020), and the local renin–angiotensin and nervous systems—endothelial cells undergo structural and functional abnormalities that accelerate keloid progression.

This review systematically examines the pivotal role of the endothelial cell system in keloid pathogenesis and progression, providing a detailed elucidation of the underlying pathological mechanisms and offering insights for the development of novel targeted therapies.

## 2 Morphological and functional alterations of VECs in keloids and the influence of environmental factors

### 2.1 Abnormal structural changes in VECs

Clinical observations indicate that keloids often appear congested owing to neovascularization and increased peripheral arterial blood flow (Eura et al., 2022). Histologically, hematoxylin and eosin (H&E) staining and CD31 immunohistochemistry confirm a high density of neovessels at the leading edge of the scar, whereas the central region is sparsely vascularized (Wen et al., 2025), suggesting an uneven distribution of vascular density and blood perfusion within the lesional tissue (Teng et al., 2022). Scanning electron microscopy reveals disordered arrangement, irregular contours, and disrupted intercellular continuity among endothelial cells in scar tissue; occlusive or stenotic changes are additionally evident in the capillary lumina (Wen et al., 2025). These pathological changes indicate significant impairment of the local blood supply, which influences keloid progression (Chen et al., 2021; Liu et al., 2016).

### 2.2 Dysfunction of VECs

During wound healing, new blood vessels and lymphatic vessels form. VEGF-A induces neovascularization with assistance from VEGF-B, VEGF-C and VEGF-D regulate lymphangiogenesis, and placental growth factor (PlGF) also contributes to the regulation of angiogenesis (Dvorak, 2021). Angiogenesis initiates during the proliferative phase, generating immature, leaky, highly permeable vessels whose elevated permeability results from the direct action of VEGF (Dvorak, 2021). This constitutes an active, controlled, and critical healing strategy: the exuded, protein-rich plasma constructs a reparative scaffold and delivers abundant growth factors to activate cells and supply nutrients (Berman et al., 2017; Surasak, 2025). In the keloid microenvironment, however, uncontrolled VEGF activation critically drives structural disorganization, impaired maturation, and hyperpermeable angiogenesis—all sustained by persistently high expression levels. This ultimately leads to ineffective perfusion and persistent hypoxia, which further amplifies inflammatory and fibrotic responses, establishing a vicious cycle (Kumar and Kamalasanan, 2021).

Another pathological manifestation is altered vascular branching, characterized by marked proliferation of the vascular network within lesional tissue—increased vessel number, intricate branching, and luminal dilation—that forms characteristic dendritic vessels (Wang et al., 2025b). Despite the increased vessel number, the morphology of these vessels is often compressed, and pericyte coverage is markedly insufficient (Armulik et al., 2011). Pericytes normally maintain microvascular homeostasis by inhibiting angiogenesis and eliminating dysfunctional vessels; defects in pericyte ensheathment therefore disrupt the vascular barrier, impair perfusion, and cause abnormally increased permeability (Mäe et al., 2021).

**FIGURE 1:**
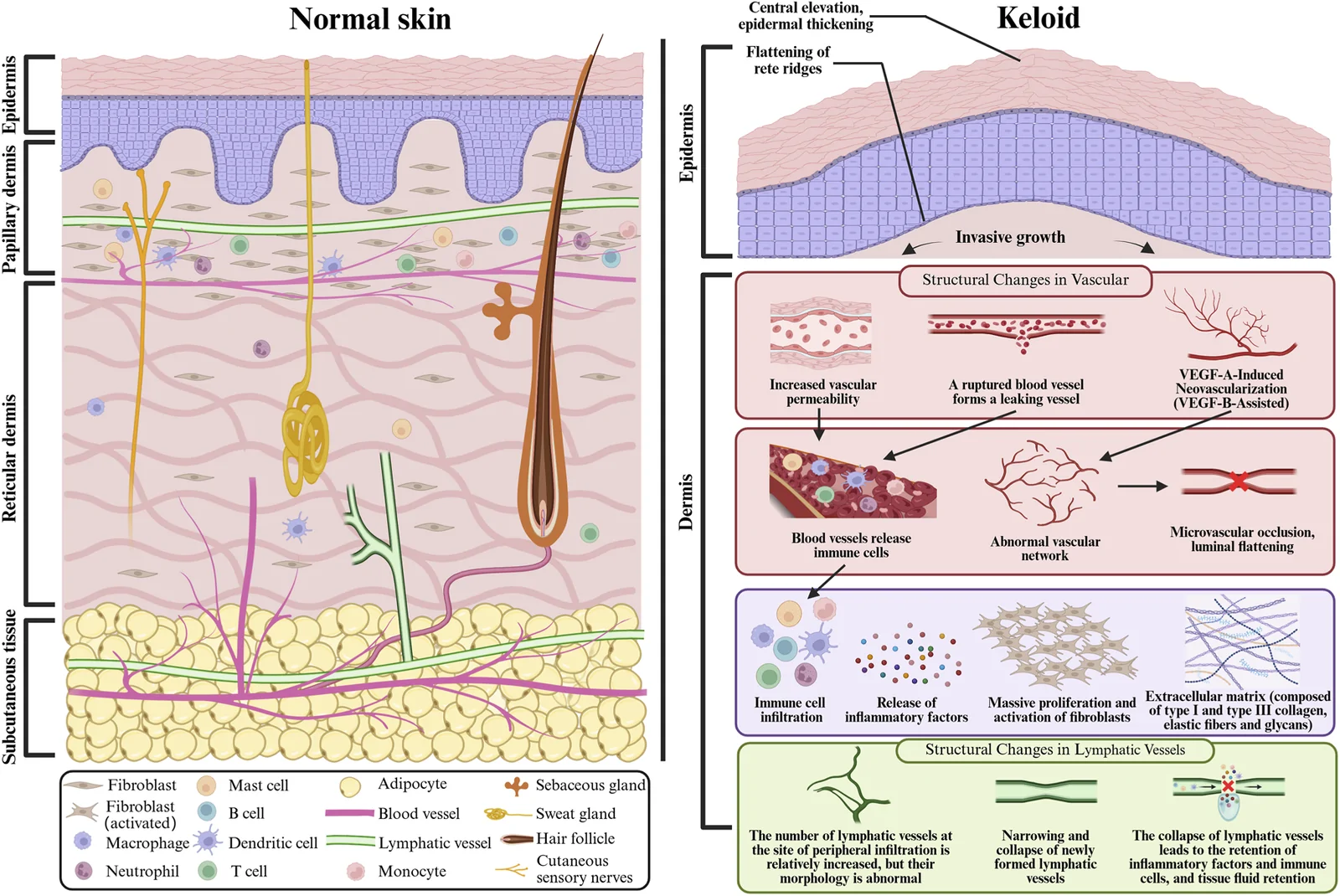
Schematic comparison of full-thickness skin structure in normal skin and keloid. Compared to normal skin, the keloid epidermis is elevated and thickened, with flattened rete ridges. In the keloid dermis, fibroblast proliferation and neovascularization are highly active, and dendritic vessels are abundant. However, these newly formed vessels are hyperpermeable, have flattened lumens, and display structural abnormalities, including leakage. Additionally, lymphatic vessels in the keloid are reduced in number, with flattened or disrupted lumens, leading to inflammation and impaired lymphatic drainage. Created in https://BioRender.com.

Endothelial dysfunctions (EDs), characterized by angiogenic disturbances, reduced capillary density, microvascular occlusion, and luminal flattening, act synergistically to induce local tissue hypoxia and promote metabolic abnormalities (Zheng et al., 2021). This plays a pivotal role in scar pathogenesis, sustaining persistent inflammatory responses and excessive ECM deposition. It should be noted that “luminal flattening” was quantitatively defined by Kurokawa et al. (2010) by measuring the ratio of the long axis to the short axis of capillary lumens in CD31-immunostained sections; a decrease in this ratio indicated luminal flattening (Kurokawa et al., 2010). Furthermore, electron microscopy revealed that occluded or narrowed vascular lumens were primarily observed in the central regions of keloids (Eura et al., 2022). Notably, abnormally increased vascular permeability is the most prominent functional characteristic of keloids, establishing a vicious cycle of inflammation and vascular dysfunction (Huang and Ogawa, 2020; Saijo et al., 2024) (Figure 1).

### 2.3 Hypoxic microenvironment

Immunohistochemistry and hypoxia probe techniques confirm a significant oxygen partial pressure gradient within keloid tissue, with the central region exhibiting a hypoxic state (Okuno et al., 2018). This hypoxic microenvironment arises from the interplay of restricted oxygen supply and increased oxygen consumption. Keloids pathologically feature excessive deposition of ECM—particularly collagen fibers—which encases the tissue and creates low oxygen levels (Qiu et al., 2023). Concurrently, localized fibroblasts undergo excessive proliferation with vigorous anabolic activity, significantly increasing tissue oxygen consumption. This mismatch between high metabolic demand and low oxygen delivery constitutes the structural basis for the hypoxic microenvironment (Wang et al., 2021).

In keloids, HIF-1α, a central transcription factor for hypoxia sensing and response, is overexpressed in VECs in a hypoxia-dependent manner and contributes to angiogenesis and functional regulation (Wu et al., 2017). It drives metabolic reprogramming by upregulating glycolysis-related enzymes, shifting energy metabolism from oxidative phosphorylation to anaerobic glycolysis and establishing a Warburg-like metabolic phenotype (Kim et al., 2006). Additionally, HIF-1α inhibits mitochondrial oxidative phosphorylation and reduces oxygen consumption, thereby exacerbating metabolic dysregulation within the local microenvironment (Wang et al., 2021). Furthermore, HIF-1α activates and drives VEGF transcription, promotes pathological angiogenesis, and mediates resistance to radiotherapy and anti-angiogenic therapies (Liu et al., 2016). However, these newly formed vessels often exhibit structural abnormalities and functional defects; rather than improving tissue perfusion, they perpetuate a vicious cycle of hypoxia.

**FIGURE 2:**
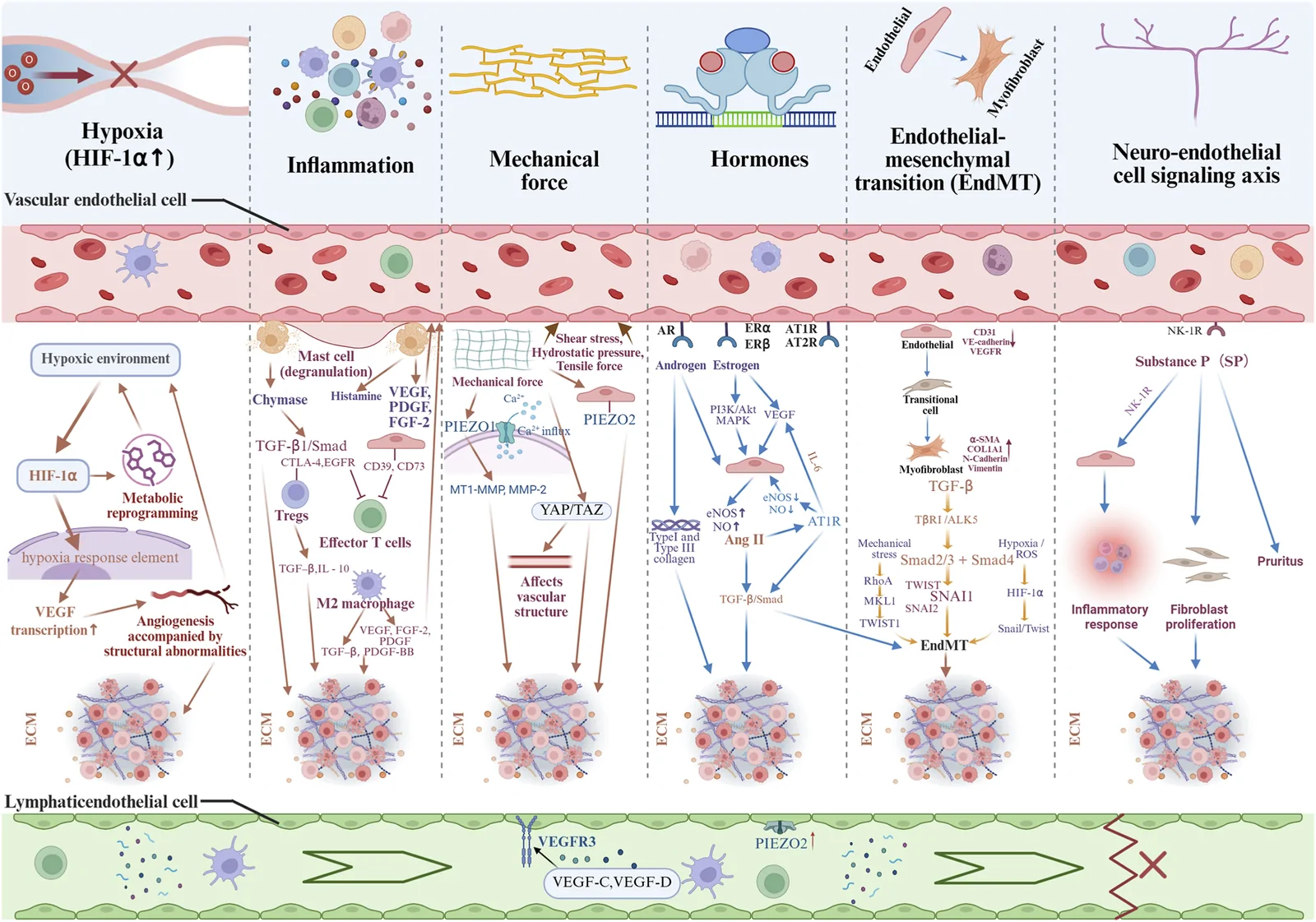
Signaling pathways of vascular and lymphatic endothelial cells in keloids regulated by diverse factors. Hypoxia, primarily through HIF-1α, induces metabolic reprogramming and vascular structural and functional abnormalities, thereby promoting fibrosis. Immune cells (predominantly M2 macrophages, Tregs, and mast cells) and associated inflammatory factors mediate chronic inflammation and fibrosis Mechanical forces (shear stress, hydrostatic pressure, and tensile force) alter vascular structure and promote extracellular matrix (ECM) production. Hormones (sex hormones and renin–angiotensin system components) modulate vascular endothelial cell function and promote the deposition of type I and type III collagen. Endothelial-to-mesenchymal transition (EndMT) is depicted as a convergent mechanism driven by these factors to augment fibrosis. Substance P acts on endothelial cells via the NK-1 receptor (NK-1R) to promote inflammation, stimulates fibroblast proliferation, and mediates pruritus. The blue arrows represent inferential pathways, while the others are supported by direct evidence regarding keloids. Created in https://BioRender.com.

Mechanistically, the VEGF gene promoter contains a hypoxia response element (HRE); upon translocation into the nucleus and binding to the HRE, HIF-1α markedly enhances VEGF mRNA transcription, establishing a hypoxia-driven signaling cascade (Zhang et al., 2025). VEGF promotes the proliferation, migration, and lumen formation of VECs in a dose-dependent manner, driving nascent capillary sprouting (Rodrigues et al., 2019). In early wound healing, local hypoxia acts as a physiological signal, recruiting inflammatory cells such as macrophages and neutrophils to secrete VEGF, thereby initiating angiogenesis, promoting granulation tissue formation and ECM remodeling, and ultimately facilitating tissue repair (Qiu et al., 2023). However, during the pathological progression of keloids, this signaling pathway is persistently hyperactivated; the persistent hypoxic microenvironment prevents the timely shutdown of the HIF-1α/VEGF axis (Wang et al., 2022). Consequently, uncontrolled angiogenesis and imbalanced ECM deposition lead to pathological scar formation (Figure 2).

### 2.4 The chronic inflammatory microenvironment in keloids

Keloids are also considered a chronic inflammatory skin disease characterized by persistent, low-grade local inflammation (Rodrigues et al., 2019). The interaction between VECs and immune cells promotes ECM deposition and inflammation progression (Fujita et al., 2019; Chen et al., 2019). In early injury, VECs release cytokines to recruit immune cells; neutrophils arrive first to prevent infection, followed by macrophages that clear debris (Berman et al., 2017). Keloid tissue exhibits auto-inflammatory -like characteristics, including upregulated expression of inflammatory cytokines (IL-1α, IL-1β, IL-6, TNF, and TGF-β) and infiltration of various immune cells (Jiao et al., 2015); among these, macrophages, T cells, and mast cells play particularly critical roles.

#### 2.4.1 Differential roles and coordination of key immune cell types

Within the inflammatory microenvironment of keloids, regulatory T cells (Tregs), mast cells and macrophages, exhibit distinct yet highly coordinated roles in modulating endothelial function and driving fibrosis.

Tregs (CD4^+^CD25^+^Foxp3^+^CD127^-^ T cells) are significantly increased in keloids, whereas the proportions of conventional CD4^+^ and CD8^+^ T cells are relatively decreased (Guit et al., 1986; Xu et al., 2022). Based on a mechanistic hypothesis derived from studies in other inflammatory contexts, VECs may synergize with Tregs by expressing ectonucleotidases CD39 and CD73, which hydrolyze pro-inflammatory ATP into immunosuppressive adenosine, thereby downregulating MHC-II expression and attenuating effector T-cell responses (Li et al., 2025). Tregs can crawl persistently on inflamed endothelial surfaces, and microvascular endothelial cells serve as their target for anti-inflammatory actions within the skin (Snelgrove et al., 2019; Norman et al., 2025). Although Tregs are intrinsically equipped to limit inflammation and promote tissue repair, their accumulation in keloids may contribute to fibrosis by suppressing anti-fibrotic immune surveillance (e.g., via IL-10 and TGF-β secretion), rather than through the establishment of a globally immunosuppressive microenvironment.

Mast cells are prominent effector cells whose increased numbers and activated degranulation state are defining pathological features of keloid tissue (Dong et al., 2014). Unlike the sustained modulation by macrophages and Tregs, mast cells act as rapid responders. They are frequently located around blood vessels and nerves, enabling immediate reactions to injury signals and direct regulation of local vascular responses (Dong et al., 2014). Mast cells and endothelial cells form heterotypic gap junctions, providing a structural basis for rapid electrochemical signal transmission (Au et al., 2026; Wilgus and Wulff, 2014). Following activation, mast cells release the mast cell-specific protease chymase, which activates the TGF-β1/Smad signaling axis to drive fibrosis (Dong et al., 2014). Simultaneously, they degranulate and secrete key growth factors (VEGF, PDGF, FGF-2) that stimulate endothelial cell proliferation, migration, and tube formation, as well as inflammatory mediators (histamine, IL-6, IL-8) that increase vascular permeability and induce vasodilation, thereby promoting inflammatory cell recruitment and exacerbating the fibrotic response (Komi et al., 2020; Artuc et al., 1999). Importantly, mast cell numbers and activation status positively correlate with scar vascular density, underscoring their close functional coupling with VECs (Ammendola et al., 2014).

Macrophages are key orchestrators of the fibrotic response. They originate from circulating monocytes that extravasate from the vasculature and subsequently recruit additional macrophages through autocrine release of MCP-1 and LTB4 (Peña and Martin, 2024). Macrophages exhibit remarkable phenotypic plasticity, with M1 macrophages (expressing IL-12 and iNOS) exerting pro-inflammatory effects, whereas M2 macrophages (expressing IL-10 and TGF-β) promote tissue repair and fibrosis (Jin et al., 2018). M1 macrophages appear primarily during early inflammation, activated by DAMPs, IFN-γ, and TNF, and secrete pro-inflammatory cytokines (TNF-α, IL-1β, IL-6) and reactive oxygen species (ROS) to eliminate pathogens (Eming et al., 2021; Willenborg et al., 2021; Willenborg et al., 2022). In the keloid microenvironment, however, persistent mechanical tension and tissue hypoxia drive extensive and sustained M2 polarization, maintaining their pro-fibrotic functions (Zhou et al., 2022; Dai et al., 2024). M2 macrophages secrete large amounts of TGF-β1 and PDGF-BB, which induce keloid fibroblasts to express α-SMA and synthesize type I and III collagen (Wang et al., 2024; Zhang et al., 2023). Concurrently, M2 macrophage-derived TGF-β can induce EndMT, further expanding the fibroblast-like cell population, while their secretion of VEGF, FGF-2, and PDGF promotes aberrant neovascularization that paradoxically results in local hypoperfusion and metabolic imbalance (Wu et al., 2026; Bai et al., 2018).

In summary, while macrophages drive fibrosis through sustained growth factor-mediated fibroblast activation and EndMT, Tregs contribute by subverting anti-fibrotic immune surveillance, and mast cells amplify the response through rapid vascular-permeability changes and paracrine signaling. Despite these mechanistic differences, all three cell types converge synergistically to sustain VEC dysfunction, perpetuate chronic inflammation, and consolidate the fibrotic phenotype of keloids.

#### 2.4.2 Contrasting M1/M2 macrophage dynamics in keloids versus normal wound healing

A hallmark distinction between normal tissue repair and keloid pathogenesis lies in the spatiotemporal dynamics of M1/M2 macrophage polarization. During normal wound healing, early-infiltrating M1 macrophages undergo an orderly and time-restricted transition into M2 macrophages under the regulation of a series of signals, thereby orchestrating the shift from pro-inflammatory defense to pro-resolving tissue repair (Xu et al., 2020). In this physiological context, M2 macrophages perform essential pro-resolution functions—including efficient clearance of apoptotic neutrophils, mitigation of secondary tissue damage, and a metabolic switch from glycolysis to oxidative phosphorylation—and their presence is self-limited, ensuring timely termination of the repair process (Zhou et al., 2022; Murray, 2017).

In striking contrast, keloids exhibit a profound disruption of this orderly M1 to M2 transition. Persistent mechanical tension and tissue hypoxia within the lesional microenvironment drive extensive and sustained M2 polarization, while simultaneously preventing the physiological downregulation of the M1 phenotype (Zhou et al., 2022; Dai et al., 2024). Unlike the transient, self-limiting M2 presence in normal wounds, M2 macrophages in keloids persist aberrantly. Consequently, the physiological functions of M2 macrophages—which in normal healing are carefully orchestrated to resolve inflammation and remodel ECM—are not only prolonged but also pathologically amplified. Rather than promoting scar maturation, this persistent M2 overabundance fundamentally subverts the repair process, transforming the macrophage population from a beneficial, repair-orchestrating force into a chronic driver of pathological fibrosis. The sustained M2 dominance further exacerbates VECs dysfunction by promoting dysregulated angiogenesis and perpetuating local hypoxia, thereby establishing a self-amplifying feed-forward loop that sustains the fibrotic microenvironment and distinguishes keloid pathology from normal physiological scar maturation.

### 2.5 Mechanical force

Mechanical forces within the keloid microenvironment arise from multiple sources, including skin tension and stretch, ECM stiffness, compression, and shear stress (Wen et al., 2025). Accordingly, keloids preferentially develop at sites subjected to mechanical tension—such as the shoulders and anterior chest—and their distinctive shapes (e.g., butterfly, crab-claw, and dumbbell) reflect the direction of these forces (Tokuyama et al., 2015; Ogawa et al., 2012).

Mechanotransduction pathways in VECs are highly complex and play a central role in sensing and responding to mechanical stimuli, thereby regulating key pathological processes such as angiogenesis and vascular permeability. VECs are intrinsically mechanosensitive and capable of detecting a variety of hemodynamic forces, including shear stress, hydrostatic pressure, and cyclic stretch (Hsieh et al., 2014). Prolonged exposure to these forces induces endothelial dysfunction, which is characterized by basement membrane disruption, widening of intercellular junctions, and dysregulated ion homeostasis, particularly involving calcium influx and potassium efflux (Huang et al., 2017).

Mechanically activated (MA) cation channels, particularly PIEZO1 and PIEZO2, are critical mediators of cellular responses to mechanical stress (Coste et al., 2010). In endothelial cells, PIEZO1 responds to mechanical stimuli such as fluid shear stress and elevated microvascular pressure; functional activation triggers Ca^2+^ influx, which, through a positive feedback loop, further enhances PIEZO1 membrane density (Rennekampff et al., 2024). As a key regulator of vascular biology, PIEZO1 is involved in inflammation, vascular development, and endothelial homeostasis (Kang et al., 2019). During angiogenesis, PIEZO1 promotes vascular sprouting by activating membrane-type matrix metalloproteinase-1 (MT1-MMP) and matrix metalloproteinase-2 (MMP-2) (Lee et al., 2013).

Notably, PIEZO1 and PIEZO2 exhibit markedly distinct expression patterns within the keloid microenvironment. PIEZO1 is broadly expressed across multiple cell types, including VECs, fibroblasts, and immune cells, functioning as a ubiquitous mechanosensor (Rennekampff et al., 2024). In contrast, PIEZO2 is primarily highly expressed in vascular endothelial cells, lymphatic endothelial cells, and specific subpopulations of fibroblasts (heightened expression of collagen genes, including COL1A1, COL1A2, COL3A1, and COL6A2) within keloids (Akita et al., 2025). This cell-type-specific distribution suggests that while PIEZO1 mediates general mechanotransduction across diverse cell populations, PIEZO2 serves as a more specialized mechanosensor, particularly linking mechanical stimuli to collagen-producing fibroblasts and endothelial cells within the keloid niche.

The clinical relevance of PIEZO2 expression is underscored by its strong correlation with key fibrotic markers and keloid recurrence. PIEZO2 expression levels correlate significantly with COL1A2 (r = 0.9252, 95% CI 0.8474–0.9641, p < 0.001) and POSTN (r = 0.9118, 95% CI 0.8213–0.9575, p < 0.001), two key extracellular matrix components in keloid fibrosis (Akita et al., 2025). More importantly, PIEZO2 expression is significantly elevated in recurrent keloids compared with non-recurrent lesions (p = 0.032), and patients with higher PIEZO2 expression experience a significantly shorter time to recurrence after keloidectomy (4/5 vs. 0/5, p = 0.047). The same study further showed that PIEZO2-positive cells and inflammatory cells co-localize in perivascular active areas of keloid tissue, implicating PIEZO2 in the establishment and maintenance of a perivascular microinflammatory environment. Collectively, these findings position PIEZO2 not merely as a mechanotransducer but also as a potential prognostic biomarker for keloid recurrence and a promising therapeutic target.

Beyond the direct effects on endothelial cells, mechanically activated fibroblasts also reciprocally induce endothelial activation. Zhang et al. identified a CD74^+^ fibroblast subset in keloids that exhibits pro-angiogenic and stretch-induced proliferative capacities (Zhang et al., 2024). Immunostaining localized these fibroblasts predominantly to the active periphery of keloids, in close proximity to vasculature. Mechanistically, mechanical stretching triggers PIEZO1-mediated Ca^2+^ influx in CD74^+^ fibroblasts, activating ERK/AKT signaling and promoting angiogenesis. Thus, mechanical forces directly activate endothelial cells while simultaneously stimulating a fibroblast subset that propagates pro-angiogenic signals back to the endothelium, establishing a reciprocal mechano-signaling loop. Collectively, the endothelial-fibroblast mechanical interaction is a bidirectional process that perpetuates the fibro-proliferative and angiogenic phenotype of keloids.

Yes-associated protein (YAP) and transcriptional coactivator with PDZ-binding motif (TAZ) are key mechanosensitive transcriptional regulators that convert physical cues into gene expression programs (Fang et al., 2025). YAP and TAZ activate actin cytoskeleton remodeling and, by upregulating MYC signaling, coordinate endothelial cell proliferation and metabolism (Kim et al., 2017). This study further revealed that YAP/TAZ are responsible for regulating the integrity of endothelial cell junctions during sprouting angiogenesis, as well as during the development and maturation of the vascular barrier. Mechanical stress stimulates Neuropilin-1 (NRP1) expression and releases YAP from NRP1, promoting YAP nuclear translocation (Li et al., 2023). This YAP activation promotes cell proliferation, inhibits apoptosis, drives excessive ECM deposition, and ultimately contributes to scar formation (Chu and Quan, 2024).

Different types of mechanical forces exert distinct effects on endothelial cells. Laminar shear stress, for example, can induce endothelial alignment and morphological remodeling and promote angiogenesis through nitric oxide (NO) production and vascular endothelial growth factor (VEGF) signaling (Glen et al., 2012). Hydrostatic pressure stimulates endothelial NO production in a Ca^2+^-dependent manner (Kaestle et al., 2007). Cyclic tensile strain induces cytoskeletal reorganization, leading to endothelial alignment perpendicular to the direction of stretch, a process regulated by cytoskeletal tension and focal adhesion dynamics (Shao et al., 2014).

Furthermore, cyclic stretching upregulates neuropeptides such as substance P (SP), which, via mast cells, enhances VEGF secretion and increases vascular endothelial permeability (Takagaki et al., 2020). Concurrently, mechanical stretching stimulates dermal microvessels to release endothelin-1 (ET-1), which promotes fibroblast differentiation into myofibroblasts, collagen synthesis, and enhanced contractility by activating the RhoA/Rho kinase pathway (Kawai et al., 2023; Kiya et al., 2017).

### 2.6 EndMT

Within the keloid microenvironment, endothelial homeostasis is disrupted, thereby promoting EndMT. This process involves the transdifferentiation of endothelial cells into mesenchymal or fibroblast-like cells and is characterized by the loss of intercellular junctions, disruption of the characteristic cobblestone-like morphology, and cytoskeletal reorganization (Piera-Velazquez and Jimenez, 2019; Alvandi and Bischoff, 2021). EndMT represents a critical mechanism by which VECs give rise to α-SMA^+^ myofibroblasts, thereby establishing a direct link between vascular dysfunction and keloid fibrosis (Fang et al., 2025). Importantly, the evidence for EndMT in keloids warrants careful interpretation. While immunofluorescence co-localization studies have demonstrated the presence of CD31/vimentin double-positive cells in keloid tissue, these findings indicate the existence of intermediate cells undergoing phenotypic transition (Lee et al., 2015). However, to date, no lineage-tracing study has been performed in keloids to definitively prove that endothelial cells give rise to myofibroblasts *in vivo*.

Depending on the nature of the initiating stimulus, EndMT in the keloid microenvironment can be categorized into three major signaling branches: 1) mechanical stress-driven, 2) inflammation-driven, and 3) hypoxia-driven pathways. Despite their distinct upstream triggers, these pathways converge on a common set of downstream transcription factors—SNAI1 (Snail), SNAI2 (Slug), and TWIST—which orchestrate the execution of the endothelial-to-mesenchymal phenotypic switch.

#### 2.6.1 Mechanical stress-driven EndMT signaling

In the high-tension keloid microenvironment, mechanical stimuli are transduced via the integrin-cytoskeleton system, leading to RhoA-mediated F-actin polymerization and a reduction in intracellular G-actin (Ma et al., 2020). This conformational change releases MKL1 (megakaryoblastic leukemia 1) from cytoplasmic sequestration; MKL1 then translocates to the nucleus, where it cooperates with serum response factor (SRF) to upregulate TWIST1 expression, suppress endothelial markers (VE-cadherin, CD31), and induce mesenchymal genes (α-SMA), thereby driving EndMT (Miralles et al., 2003). Mechanical stimuli, including shear stress and tissue tension, also promote EndMT. Under pathological conditions, aberrant forces reprogram endothelial cells and activate Notch and TGF-β pathways, leading to SNAI1 and TWIST upregulation (Dessalles et al., 2021; Mack et al., 2017).

#### 2.6.2 Inflammation-driven EndMT signaling

Inflammatory mediators—particularly TGF-β, IL-1β, and TNF-α—act as potent inducers of EndMT (Pérez et al., 2017). The TGF-β/Smad pathway is the most extensively characterized inflammatory signaling axis in EndMT. All three TGF-β isoforms (TGF-β1, TGF-β2, TGF-β3) are capable of driving this transition (Pardali et al., 2017). TGF-β signals through type I receptors (TβRI/ALK5) and activin receptors (ALK4/7) to phosphorylate Smad2 and Smad3 (Ma et al., 2020). Phosphorylated SMAD2/3 complexes with SMAD4 and translocates to the nucleus, where it activates EndMT-associated transcription factors—SNAI1, SNAI2, and TWIST—thus establishing the TGF-β - Smad2/3 - Snail/Twist axis and promoting α-SMA and collagen expression (van Meeteren and ten Dijke, 2012; Xu and Kovacic, 2023).

The Notch pathway synergizes with TGF-β signaling in human microvessels, where Notch and TGF-β cooperatively induce Snail by recruiting Smad3 to the promoters of HEY1, HEY2, HEYL, SNAIL, and ANKRD1 (Fu et al., 2009). Snail subsequently downregulates endothelial markers (CDH5, PECAM1) and upregulates mesenchymal genes (ACTA2, FN1, COL1A1), further driving EndMT. IL-1β and TGF-β2 synergistically promote EndMT; notably, TGF-β2 is the only isoform progressively upregulated during combined IL-1β/TGF-β2 treatment (Pérez et al., 2017; Maleszewska et al., 2013). The triple combination of TGF-β, IL-1β, and TNF-α elicits an even more robust EndMT response. Additionally, the p38-MAPK pathway mediates TGF-β2-induced EndMT (Yun et al., 2020). Thus, the inflammatory milieu of keloids—enriched in TGF-β and other cytokines—provides persistent stimulation of Smad-dependent and Smad-independent EndMT pathways.

#### 2.6.3 Hypoxia-driven EndMT signaling

The hypoxic keloid microenvironment drives EndMT through multiple interconnected mechanisms. Hypoxia stabilizes HIF-1α, which directly induces transcription factors such as Twist1, Slug, and Snail, promoting the acquisition of mesenchymal phenotypes by endothelial cells (Zhang et al., 2018). Most ROS generated under hypoxic conditions are produced via the NADPH oxidase system; NOX4, a member of this family, serves as a critical downstream mediator of TGF-β-induced myofibroblast differentiation (Kim and Chang, 2025). Activation of the Nrf2/HO-1 antioxidant pathway, in contrast, inhibits EndMT, suggesting that the balance between ROS production and antioxidant defense determines the net EndMT outcome under hypoxic stress (Yun et al., 2020). These hypoxia-activated pathways reinforce and amplify the EndMT response initiated by mechanical and inflammatory stimuli, creating a permissive environment for persistent endothelial dysfunction.

#### 2.6.4 Convergence of EndMT signaling pathways

Despite their distinct upstream triggers, the three signaling branches described above converge on a common downstream effector module: the transcriptional reprogramming mediated by Snail, Slug, and Twist family transcription factors. These factors bind to E-box elements in the promoters of endothelial-specific genes (CDH5, PECAM1), repressing their expression, while simultaneously activating mesenchymal gene programs (ACTA2, FN1, COL1A1) (Xu and Kovacic, 2023; Maleszewska et al., 2013). The net effect is the loss of endothelial identity and the gain of fibroblast-like phenotypes, including α-SMA expression, enhanced contractility, and active ECM production. Moreover, these pathways do not operate independently; cross-talk among them is extensive. TGF-β can be activated downstream of mechanical stress, hypoxia amplifies TGF-β signaling by upregulating its receptors, and inflammatory cytokines further stabilize HIF-1α. This interwoven network ensures that EndMT, once initiated, is sustained by multiple reinforcing inputs.

In summary, EndMT bridges vascular alterations and fibroblast accumulation within the keloid microenvironment. Mechanical stress, inflammatory mediators, and hypoxia collectively induce EndMT through YAP/TAZ-MKL1/SRF, TGF-β/Smad-Notch, and HIF-1α/ROS pathways, respectively, all converging on Snail/Twist-mediated transcriptional reprogramming. The resultant generation of activated fibroblasts and myofibroblasts promotes collagen deposition; this process may be further amplified as ECM accumulation increases tissue stiffness, potentially triggering a feed-forward cascade that perpetuates EndMT and progressive fibrosis.

### 2.7 Hormone

Hormones play essential roles in systemic metabolism and hemodynamic regulation and are increasingly recognized as key modulators of the keloid microenvironment. VECs express a variety of hormone receptors, including those for sex hormones and components of the renin–angiotensin system (RAS) (Zhang et al., 2023). Accumulating evidence suggests that hormonal signaling contributes to keloid initiation and progression through the regulation of inflammation, vascular permeability, and EndMT.

#### 2.7.1 Sex hormones

Epidemiologically, adolescence and pregnancy are periods during which keloids are more likely to develop or worsen (Ibrahim et al., 2020; Talluri and Gurram, 2025). Accumulating evidence implicates systemic endocrine factors—particularly sex hormones (estrogens and androgens)—in the pathogenesis and progression of keloids (Yang et al., 2022). The high concordance of keloids in monozygotic twins and the marked ethnic disparities (substantially higher incidence in African and Asian populations than in Caucasians) collectively point to a strong genetic susceptibility (Greene et al., 2025). Mechanical tension and genetic susceptibility also synergistically influence scar severity (Xia et al., 2024).

At the cellular level, VECs widely express estrogen receptors (ERα, ERβ) and androgen receptors (AR); hormone binding activates downstream signaling pathways that regulate endothelial function (Ishikawa et al., 2013). Estrogen promotes endothelial cell proliferation and migration, enhances eNOS expression and NO production, and reshapes the local microenvironment through pathways such as PI3K/Akt and MAPK (Haynes et al., 2000). Estradiol (E2) also enhances vasodilation and reduces oxidative stress-induced injury by inhibiting the angiotensin pathway (Connelly et al., 2022). Moreover, estrogen enhances VEGF secretion, increases endothelial cell adhesion, proliferation, and migration, and facilitates their organization into capillary-like structures *in vitro* (Straub, 2007). Extrapolated from these observations, estrogen may exert a dual effect within the keloid microenvironment: while it can reduce inflammation and endothelial dysfunction, it concurrently promotes endothelial proliferation and migration, potentially yielding structurally and functionally defective blood and lymphatic vessels that exacerbate local fibrosis. However, direct evidence for these dual effects specifically in keloid-derived endothelial cells remains limited.

With regard to androgens, a 2020 case of a transgender male who developed eruptive facial angiofibromas following gender-affirming testosterone therapy directly demonstrated that exogenous testosterone can activate or stimulate the growth of vascular skin lesions in genetically predisposed individuals (Bubley et al., 2020). In 2024, a study using a miniature-pig pathological scar model found that testosterone treatment significantly increased fibrotic area, scar thickness, and deposition of type I and type III collagen, resulting in greater scar mechanical strength (Reiche et al., 2024). These findings confirm that androgens are an independent risk factor for abnormal scar formation, independent of chromosomal sex. Androgen receptor levels are significantly elevated in keloid tissue, especially in active keloids that present clinically with hyperemia, pain, and rapid growth (Wen et al., 2025).

#### 2.7.2 RAS

Currently, only limited data are available regarding the role of the RAS, and similarly the lymphatic vasculature, in keloid biology. The skin contains a complete RAS comprising angiotensin II (Ang II), angiotensin II type 1 receptor (AT1R), angiotensin II type 2 receptor (AT2R), and angiotensin-converting enzyme (ACE) (Steckelings et al., 2004; Dai et al., 2018). AT1R mediates pro-inflammatory, pro-fibrotic, and pro-EndMT effects, whereas AT2R exerts opposing anti-fibrotic and anti-proliferative actions (Hedayatyanfard et al., 2020).

Although most mechanistic insights into RAS signaling derive from studies in cardiovascular biology and systemic fibrosis, Ang II acts via AT1R to upregulate inflammatory factors such as IL-6, promote VEGF-driven angiogenesis, and activate the TGF-β1/CTGF axis to drive ECM deposition and inhibit tissue inhibitors of metalloproteinases (TIMPs), thereby promoting fibrosis (Hedayatyanfard et al., 2020). Furthermore, Ang II/AT1R signaling activates the ROCK pathway, inhibits endothelial nitric oxide synthase (eNOS), reduces NO bioavailability, and impairs endothelial vasodilation and barrier function, leading to endothelial dysfunction (Kinzenbaw et al., 2022; De Silva et al., 1979). Based on evidence from vascular remodeling studies, Ang II induces EndMT during vascular remodeling and injury via both canonical and non-canonical pathways. Canonically, it activates TGF-β/Smad signaling through AT1R (Yu et al., 2026). Non-canonically, it activates the NF-κB pathway via Toll-like receptor 2 (TLR2), which mediates non-hemodynamic effects of Ang II and modulates inflammatory responses (Wang JC. et al., 2025; Fukuda et al., 2019; Chung et al., 2016). Additionally, a high-glucose environment directly stimulates endothelial cells to produce Ang II, promoting EndMT through autocrine and/or paracrine mechanisms (Tang et al., 2010). Moreover, Ang II-induced inflammatory cytokines (TNF-α, IL-6, IL-1β) further promote EndMT via paracrine positive feedback within the inflammatory microenvironment (Lin et al., 2020). It is plausible that analogous mechanisms operate in keloids, given that VECs express AT1R and keloid tissue exhibits elevated RAS components; however, direct experimental confirmation in keloid-derived endothelial cells is currently lacking.

### 2.8 Nervous system

The concept of a direct nerve–endothelial cell signaling axis posits that, following skin injury, sensory nerve fibers release neuropeptides such as SP that directly modulate endothelial cell responses (Yu et al., 2026). Conversely, neurotrophins secreted by endothelial cells also influence nerve fiber regeneration (Ward et al., 2011).

SP is among the most extensively studied neuropeptides in this context; studies in wound healing and neurogenic inflammation have shown that SP directly induces angiogenesis and promotes inflammatory responses (Scott et al., 2007). SP promotes proliferation and inhibits apoptosis of keloid fibroblasts, with effects more pronounced than those on fibroblasts from hypertrophic scars or normal skin (Jing et al., 2010). Elevated SP activity directly correlates with pruritus, one of the most distressing symptoms of keloids (Aerts et al., 2022).

The primary pro-fibrotic mechanism of SP is thought to be mediated through binding to its high-affinity receptor, NK-1R, which is expressed on both neuronal cells and VECs (Muñoz et al., 2010; Hutter et al., 2005). Drawing on evidence from vascular biology, SP binding to NK-1R on endothelial cells directly activates these cells (Muñoz et al., 2010; Hutter et al., 2005). Activated endothelial cells promote vasodilation and increase vascular permeability; this not only directly contributes to scar hypertrophy and erythema but also causes extravasation of plasma proteins, supplying the necessary matrix components for fibrosis (Akaishi et al., 2008). SP further enhances angiogenesis by mobilizing endothelial progenitor cells (EPCs) and promoting their differentiation (Um et al., 2016). Although these SP-mediated effects on endothelial cells have been well characterized in other vascular contexts, direct evidence for this signaling axis specifically in keloid endothelial cells remains to be established.

### 2.9 Synergistic interplay of microenvironmental stimuli

Although discussed separately, hypoxia, chronic inflammation, and mechanical forces do not operate in isolation but converge into a self-perpetuating positive feedback network within the keloid microenvironment. Hypoxia, driven by the HIF-1α/VEGF axis, induces pathological angiogenesis and vascular hyperpermeability, leading to plasma extravasation, tissue edema, and inflammatory cell infiltration. These infiltrating immune cells secrete pro-inflammatory cytokines (TNF-α, IL-1β, TGF-β) that directly promote EndMT via Smad-dependent and non-canonical pathways. Concurrently, mechanical tension—transduced through PIEZO1/2 channels and the YAP/TAZ axis—synergizes with TGF-β signaling to upregulate EndMT-related transcription factors (SNAI1, TWIST) and enhance ECM production, while also reinforcing angiogenesis and neurogenic inflammation through VEGF and SP upregulation. These three stimuli establish multiple feed-forward loops: hypoxia amplifies inflammation via HIF-1α-dependent cytokine transcription; inflammation increases tissue stiffness through ECM deposition, thereby augmenting mechanical stress; and mechanical stretch further upregulates inflammatory mediators and hypoxia-inducible factors. Hormonal signals (sex hormones and RAS) and neuropeptides act as contextual modulators that fine-tune endothelial reactivity and barrier permeability. Collectively, this interlaced network drives progressive endothelial dysfunction, sustains chronic inflammation and hypoxia, amplifies EndMT, and ultimately consolidates the fibrotic phenotype of keloids, highlighting the need for combination therapies that disrupt multiple nodes of this vicious cycle.

## 3 LECs

As integral components of the vascular system, LECs form lymphatic capillaries and collecting vessels, maintaining tissue fluid homeostasis and immune surveillance by clearing proteins and inflammatory mediators and facilitating immune cell trafficking (e.g., dendritic cells and T cells) (Vaahtomeri et al., 2017; Schwager and Detmar, 2019). Consequently, lymphatic drainage integrity is critical for limiting inflammation and stabilizing the local microenvironment (Alitalo, 2011).

In patients who respond to therapy, mid-dermal lymphatic vessels increase significantly after scar treatment (Komulainen et al., 2025). Following injury, macrophage-derived signals drive lymphatic vessel repair analogously to blood vessel repair; new lymphatic vessels arise from the dilation of pre-existing main vessels (Kataru et al., 2009). Prostaglandin E2 receptors EP3 and EP4, and receptor activity-modifying protein 1 (RAMP1), have been reported as potential mediators of lymphangiogenesis; however, the most widely recognized mediators of lymphatic sprouting remain VEGF receptor 3 (VEGFR3) and its ligands VEGF-C and VEGF-D (Kurashige et al., 2014; Hosono et al., 2016). Overexpression of VEGF-C increases lymphatic density, accelerating edema clearance during skin inflammation, whereas inhibition of lymphangiogenesis delays edema resolution (Peña and Martin, 2024; Huggenberger et al., 2011; Kataru et al., 2009).

Excessive ECM deposition in keloids mechanically compresses blood and lymphatic vessels, compromising luminal structure and fluid outflow; progressive fibrosis further exacerbates lymphatic drainage impairment (Eura et al., 2022). When lymphatic drainage is impaired, interstitial fluid accumulates, causing persistent edema that entrains plasma proteins and inflammatory mediators, thereby altering the extracellular environment and local osmotic pressure (Cc et al., 2023). Moreover, increased interstitial fluid extends the oxygen diffusion distance, inducing local hypoxia; this hypoxia activates inflammatory and fibrotic signaling pathways, establishing a self-amplifying positive feedback loop (Wen et al., 2025). Recent studies indicate that PIEZO2 expression is upregulated in LECs, suggesting enhanced mechanosensory responses (Akita et al., 2025). Furthermore, the lymphatic system modulates immune cell retention; the accumulation of macrophages and T cells releases cytokines and growth factors that further activate fibroblasts and influence endothelial cell function (Cc et al., 2023).

### 3.1 Molecular mechanisms of LEC dysfunction: EndMT, mechanotransduction, and immune crosstalk

Beyond these biomechanical consequences, LECs themselves undergo profound molecular alterations that actively contribute to keloid pathogenesis. First, LECs can undergo endothelial-to-mesenchymal transition (EndMT)—a process well-characterized in blood vascular endothelial cells. In systemic sclerosis, LECs in intermediate stages of Ly-EndMT (coexpressing LYVE-1 and α-SMA) are found exclusively in fibrotic skin, and culture with TGF-β1 induces downregulation of LEC markers and upregulation of myofibroblast markers (Rosa et al., 2023). Both TGF-β and TNF-α synergistically drive LEC EndMT via activation of Activin signals and establishment of an autocrine amplification loop. In the keloid context, single-cell RNA sequencing has revealed that endothelial cells participate in EndMT, suggesting LECs may serve as an additional source of profibrotic myofibroblasts. Second, the mechanically activated cation channel PIEZO2 is significantly upregulated in both vascular and lymphatic endothelial cells within keloid tissue. PIEZO2 expression shows strong correlation with COL1A2 and POSTN, and is significantly higher in recurrent keloids than in non-recurrent ones, directly linking LEC mechanosensitivity to keloid fibrosis severity and recurrence risk (Akita et al., 2025). Third, studies using lymphedema models have revealed that LECs actively modulate immune responses via sphingosine-1-phosphate (S1P) signaling; reduced LEC S1P signaling enhances LEC adhesion and amplifies pathogenic CD4^+^ T cell responses, exacerbating lymphatic insufficiency and tissue inflammation (Kim et al., 2023). Collectively, these mechanisms—Ly-EndMT, PIEZO2-mediated mechanotransduction, and LEC-immune cell crosstalk—converge to drive LEC dysfunction, perpetuating edema, chronic inflammation, and fibrosis.

### 3.2 LEC dysfunction as a driver of edema and persistent inflammation

The molecular mechanisms outlined above establish a self-perpetuating pathological cascade in keloids. Mechanical compression from excessive ECM deposition impairs lymphatic outflow, while PIEZO2 activation in LECs under chronic tension further compromises lymphatic endothelial integrity. The resulting lymphatic insufficiency causes interstitial fluid accumulation and edema, which entrains plasma proteins and inflammatory mediators, perpetuating local inflammation (Cc et al., 2023). Concurrently, TGF-β and TNF-α within the inflammatory milieu drive Ly-EndMT, converting LECs into myofibroblast-like cells that contribute directly to ECM production and fibrosis. The sustained inflammatory state, further amplified by aberrant S1P signaling and chemokine-mediated immune cell recruitment, establishes a feed-forward loop that progressively worsens lymphatic function. Unlike the transient and self-limiting lymphatic responses seen in normal wound healing, LEC dysfunction in keloids is chronic and progressive, actively driving the transition from acute inflammation to persistent fibrosis (Wen et al., 2025).

## 4 Current status of treatment

### 4.1 Anti-angiogenic therapy

Given the critical role of VEGF in keloid pathogenesis and progression, medications with anti-vascular and anti-angiogenic properties have been investigated. 5-Fluorouracil (5-FU), a pyrimidine analog antimetabolite, demonstrates significant efficacy against keloids, markedly improving scar appearance and reducing recurrence (Shah et al., 2016; King et al., 2024). The first human clinical trial confirmed that nano-5-FU effectively reduces scar height and vascularization, with exceptional and stable efficacy and significantly fewer local side effects (He et al., 2023). Compared with free 5-FU, liposomal 5-FU (5-FU-Lip) significantly inhibits proliferation and microvessel formation of human umbilical vein endothelial cells (HUVECs) (Li Y. et al., 2024). A randomized controlled trial confirmed that, in patients responding to intralesional injection therapy (triamcinolone acetonide [TAC] or 5-fluorouracil [5-FU]), the mid-dermis of keloids exhibited a significant increase in mature blood and lymphatic vessels post-treatment (Komulainen et al., 2025). Additionally, TAC is more likely to induce telangiectasia than 5-FU, 5-FU plus TAC, or bleomycin (Zhuang et al., 2021). Bevacizumab is an effective and safe adjunctive therapy that, combined with TAC, further reduces keloid height (Shahmoradi et al., 2024).

### 4.2 Targeting EndMT

Because EndMT contributes to scar formation and vascular abnormalities, targeting TGF-β represents a promising therapeutic strategy. The TGF-β3 isoform has been evaluated in clinical trials (Penn et al., 2012). Recombinant TGF-β3 (avotermin) was developed as an anti-scarring agent and improved scar appearance in several Phase I/II randomized controlled studies (Ferguson et al., 2009). Phase I studies in healthy volunteers and a small-incision model demonstrated a favorable safety profile after local injection, no significant systemic adverse reactions, and a trend toward improved scar appearance (Occleston et al., 2008). A Phase II multicenter RCT further validated efficacy: local injection at wound closure or early postoperatively improved scar flatness, color, and overall aesthetic appearance compared with placebo, particularly in low-tension areas (Ferguson et al., 2009). However, Phase III trials failed to replicate these benefits, with no statistically significant therapeutic advantage, leading to termination of development (Occleston et al., 2011). The failure of avotermin in Phase III trials is instructive and warrants in-depth analysis beyond the mere outcome. A critical contributing factor was a change in the immunoassay standard used to quantify avotermin during clinical development. Specifically, the reference standard was switched from NIBSC 98/608 to lot 205–0,505-005; the new standard exhibited only approximately 50% of the immunoreactivity of the original, resulting in a twofold overestimation of TGF-β3 concentration and, consequently, a 50% lower actual dose administered in Phase III trials compared with the Phase I/II studies (Little et al., 2012). This dosing discrepancy likely undermined the ability to achieve the therapeutic efficacy observed in earlier trials. Beyond this methodological issue, the failure also reflects the inherent complexity of the keloid microenvironment, which is characterized by persistent hypoxia, chronic inflammation, and aberrant mechanical stress. Unlike the relatively controlled settings of early-phase trials, the heterogeneous and dynamically changing pathological milieu of keloids may have counteracted the anti-scarring effects of exogenous TGF-β3, as the sustained presence of pro-fibrotic signals (e.g., TGF-β1, HIF-1α, inflammatory cytokines) could overwhelm or modify the intended therapeutic action (Durani et al., 2008). Additionally, the timing and frequency of administration—avotermin was administered at the time of wound closure and again 24 h later—may not have adequately addressed the prolonged nature of the fibrotic cascade in keloids. Collectively, these insights underscore that the clinical translation of EndMT-targeted therapies must account not only for target engagement but also for the broader microenvironmental context, dosing precision, and treatment timing. Overall, EndMT-targeted therapies remain exploratory, and their clinical translational value awaits further validation.

### 4.3 Targeting the hypoxic microenvironment (HIF-1α)

HIF-1α drives aberrant angiogenesis and endothelial cell activation by promoting transcription of target genes such as VEGF; therefore, targeting HIF-1α or ameliorating local hypoxia is a potential therapeutic strategy (Kang et al., 2020). Preliminary laboratory studies have shown that HIF-1α inhibitors (e.g., CAY10585) significantly reduce collagen secretion (Kang et al., 2020). Resveratrol downregulates HIF-1α to inhibit fibroblast proliferation and promote apoptosis (Si et al., 2020), and quercetin inhibits HIF-1α expression via the PI3K/Akt pathway (Si et al., 2018). HIF-1α also contributes to radioresistance; thus, targeting HIF-1α may enhance radiotherapy. For example, 2-methoxyestradiol (2ME2), a natural estradiol metabolite, inhibits HIF-1α expression and acts as a radiosensitizer (Si et al., 2018). 2ME2 significantly increases radiation-induced fibroblast apoptosis, thereby overcoming radioresistance (Long et al., 2016).

### 4.4 Mechanical force-related therapies

Therapies targeting mechanical forces include pressure therapy, which uses specialized garments or devices (e.g., Zimmer splints or magnets) to apply continuous pressure to the skin, altering wound tension and inducing localized hypoxia (Park et al., 2011). Botulinum toxin type A (BoNT-A) is also considered a mechanical force-related therapy, as it reduces muscle tone and thereby lowers wound tension; however, its efficacy in keloids remains inconclusive, with inconsistent findings (Schlessinger et al., 2017; Cohen and Scuderi, 2017). Additionally, postoperative tension-reducing suture techniques—such as deep tension-relieving, subcutaneous buried, and tension-dispersing sutures—directly reduce mechanical tension at the incision edges during early wound healing, thereby mitigating persistent inflammatory stimulation and the risk of abnormal fibrosis, and representing a fundamental strategy for preventing hypertrophic scarring (Chen et al., 2024). Silicone sheets are also associated with mechanical forces; proposed mechanisms include reduction of skin tension, occlusion, and hydration, and they may indirectly influence tissue edema or impaired lymphatic drainage (Gold et al., 2014; Hsu et al., 2017).

### 4.5 Sex hormone-targeted therapy

Tamoxifen citrate, a non-steroidal anti-estrogen, affects fibroblast activity and ECM metabolism (Jordan, 2003; Gr et al., 2010), possibly by modulating the TGF-β/Smad signaling pathway, a critical regulator of keloid formation (Leask and Abraham, 2004). Tamoxifen has been used as adjuvant therapy for abnormal scarring; however, current clinical evidence remains limited, with most conclusions derived from *in vitro* or small-sample studies (Soares-Lopes et al., 2017). Overall, sex hormone-targeted interventions offer new avenues for scar treatment, but their efficacy and applicable patient populations require further clarification.

### 4.6 RAS-targeted therapy

Angiotensin-converting enzyme inhibitors (ACEIs) influence wound healing by reducing collagen synthesis, TGF-β1 expression, and fibroblast proliferation (Fang et al., 2018). Enalapril, a classic ACEI, inhibits the conversion of Ang I to Ang II, thereby reducing vasoconstriction, decreasing aldosterone secretion, increasing bradykinin levels, and ultimately lowering blood pressure and improving vascular endothelial function (Mohammadi et al., 2018). Promising preliminary results have been reported: a double-blind clinical trial (n = 30) demonstrated that topical enalapril significantly reduced hypertrophic scar size (Mohammadi et al., 2018); a case report observed keloid improvement after oral enalapril (Iannello et al., 2006), and another described improvement of post-burn keloids with topical captopril, including reductions in erythema, scaling, and pruritus (Ardekani et al., 2009). However, these encouraging findings must be interpreted with considerable caution. To date, no multicenter RCT has been conducted to validate the efficacy of ACEIs in keloids or hypertrophic scars; the existing evidence comprises a single small-sample trial and isolated case reports, which are susceptible to selection bias and placebo effects. Furthermore, the optimal formulation (topical versus systemic), dosing regimen, and treatment duration remain undefined, and the potential for systemic hypotension and renal impairment with oral ACEIs presents a barrier to widespread use in patients without hypertension. Given these substantial evidence gaps, RAS-targeted therapy should currently be regarded as an experimental approach rather than an established treatment option; its clinical adoption awaits validation through rigorously designed, adequately powered multicenter RCTs. Until such evidence is available, ACEIs may be considered only in refractory cases or within clinical trial settings, with careful monitoring of systemic side effects.

### 4.7 Laser therapy

Laser therapy is a widely used modality for keloid management. Devices that affect VECs include the pulsed dye laser (PDL), variable-pulse-duration frequency-doubled Nd:YAG laser, and intense pulsed light (IPL); among these, PDL is considered the gold standard for keloid treatment (Preissig et al., 2012; Forbat et al., 2017). Beyond directly disrupting abnormal vascular structures, PDL selectively targets hemoglobin, inducing vascular coagulation, endothelial damage, and vascular occlusion, thereby inhibiting scar proliferation and alleviating pruritus and erythema (Anderson and Parrish, 1983). Moreover, PDL treatment reduces VEGF expression in scar tissue, suggesting that in addition to physical destruction, it may modulate angiogenic signaling to influence endothelial cell activity (Kuo et al., 2005). Similarly, the Nd:YAG laser, with deeper tissue penetration, acts on dermal vascular endothelium to promote vasoconstriction and ameliorate local blood-supply abnormalities (Gold et al., 2010). Studies confirm that intralesional triamcinolone combined with PDL, targeting scar erythema and vasculature, achieves over 50% improvement in 70.7% of keloid cases, with moderate-to-excellent outcomes (Stephanides et al., 2011).

In summary, endothelial- and vasculature-targeted interventions offer a diverse therapeutic landscape for keloid management, characterized by distinct mechanisms, clinical evidence levels, and applicable scenarios. Anti-angiogenic agents (e.g., 5-FU, bevacizumab) and vascular-specific lasers (e.g., PDL, Nd:YAG) represent the most clinically mature modalities. Intralesional 5-FU, particularly in nano- or liposomal formulations, effectively suppresses microvessel formation and reduces scar height, making it ideal for moderate-to-severe refractory keloids. PDL remains the gold standard for hypervascular, erythematous, and pruritic keloids through selective photothermolysis of hemoglobin and downregulation of local VEGF. Upstream microenvironmental modulators—EndMT-targeted agents (e.g., avotermin) and hypoxia regulators (e.g., CAY10585, 2ME2)—offer mechanistic potential, but their translational value remains contingent on microenvironmental heterogeneity, as exemplified by the terminated Phase III avotermin trials. Mechanical-force modulators (pressure therapy, BoNT-A, tension-reducing sutures) and repurposed systemic agents (tamoxifen, ACEIs) provide promising adjunctive or preventive options by indirectly reshaping the vascular niche, though many currently rely on small-sample or anecdotal evidence. Future paradigms will likely require stratified combination protocols—such as PDL with intralesional corticosteroids/antimetabolites—to simultaneously dismantle active vascular scaffolds and suppress fibrotic progression.

### 4.8 Emerging cell- and gene-targeted therapies

Beyond the pharmacological and physical modalities discussed above, emerging basic research directions—particularly gene therapy and exosome-based strategies—are opening new frontiers for endothelially targeted keloid treatment. In the realm of gene therapy, a 2025 study demonstrated that a polylysine/sodium alginate nanocarrier co-delivering chlorogenic acid and siRNA effectively inhibits HUVEC proliferation and induces apoptosis by regulating the cell cycle (Zhang et al., 2026). Concurrently, E-selectin-targeted siRNA liposome nanoparticles have been shown to block monocyte-endothelial cell crosstalk and prevent pathological scar formation in preclinical models (Li L. et al., 2024), while bioinformatic analyses have identified lncRNA–mRNA interaction networks in keloid VECs as potential epigenetic regulatory targets (Luo et al., 2025). In the exosome arena, adipose-derived MSC exosomes carrying miR-7846–3p suppress keloid fibroblast viability and block HUVEC angiogenesis by targeting neuropilin 2 (Wu et al., 2023); engineered exosomes overexpressing miR-29a inhibit scar formation via the TGF-β2/Smad3 pathway (Yuan et al., 2021). Despite their promise, both approaches face substantial hurdles—gene therapy requires efficient and targeted delivery to endothelial cells with rigorous safety evaluation, while exosome-based therapies lack standardized production protocols and large-scale clinical validation. Nevertheless, these emerging directions hold substantial potential for expanding the therapeutic arsenal against keloids and merit close attention in future research.

## 5 Discussion

Keloid hyperemia results from increased vascularity and elevated blood flow. However, these vessels often display structural abnormalities and heterogeneous perfusion, compromising oxygen supply and generating a hypoxic microenvironment. Hypoxia upregulates HIF-1α, driving angiogenesis and metabolic reprogramming. Inflammation persists throughout scar development: through interactions with VECs, M2 macrophages, Tregs, and mast cells promote vascular regeneration and sustained fibrosis. Mechanical forces induce endothelial dysfunction characterized by increased permeability and structural alterations. Under the combined influence of these stimuli, VECs undergo EndMT, expanding the myofibroblast population and ECM accumulation. Sex hormones, the renin-angiotensin system, and neuropeptides further modulate endothelial function, while impaired lymphatic drainage leads to edema that perpetuates inflammation and hypoxia. However, the relative certainty of these mechanisms varies substantially (see evidence grading below).

Several issues warrant critical discussion. First, estrogen exerts paradoxical endothelial effects: it attenuates inflammation via NF-κB inhibition, yet simultaneously promotes VEC proliferation and VEGF secretion, yielding defective vessels that exacerbate fibrosis—complicating hormonal intervention development. Second, BoNT-A efficacy in keloids remains inconclusive, with inconsistent results across studies, likely due to variations in dosing and lesion characteristics. Third, TGF-β3 (avotermin) failed in Phase III trials despite early promise, underscoring that net fibrotic outcome reflects dynamic equilibrium among TGF-β isoforms, heavily influenced by the local microenvironment.

Current animal models—rabbit ear scars and mouse xenografts—fail to recapitulate the spontaneous, chronic, mechanically influenced nature of human keloids, lacking both genetic susceptibility and distinct biomechanical microenvironment. The multi-factorial keloid network means single-target therapies are unlikely to break the self-amplifying vicious cycle, explaining why many preclinical successes fail clinically. Additionally, LECs remain underexplored, with current understanding largely limited to biomechanical consequences.

To facilitate critical interpretation, we grade mechanisms by directness. Tier I (direct keloid evidence): hypoxia (HIF-1α confirmed in keloid tissue by immunohistochemistry/Western blot), mechanical forces (PIEZO2 upregulation in keloid VECs/LECs by single-cell analysis, with quantitative correlations to fibrotic markers and recurrence risk), and chronic inflammation (immune cell infiltration well established histologically). Tier II (plausible, extrapolated): RAS and neuropeptide pathways—while receptors are expressed in keloids, mechanistic details of Ang II-induced EndMT and SP-mediated activation derive primarily from cardiovascular and neurogenic inflammation models. Tier III (hypothetical): sex hormone pathways—epidemiological associations are robust, but direct evidence for estrogen-/androgen-mediated endothelial effects in keloid-derived VECs remains limited.

Several endothelial molecules hold promise as recurrence predictors. HIF-1α and CD31 are elevated in recurrent keloid tissues. More compellingly, PIEZO2 correlates strongly with COL1A2 and POSTN, and higher expression predicts shorter recurrence-free survival after keloidectomy, positioning it as a promising candidate for post-surgical risk stratification. Integrating HIF-1α, CD31, and PIEZO2 profiling could enable precise identification of high-risk patients and guide adjuvant therapy intensity.

Multi-target combination therapies addressing angiogenesis, mechanical stress, and lymphatic dysfunction simultaneously are likely superior to single agents. Lymphatic-targeted strategies (VEGF-C/VEGFR3 agonists) warrant investigation to restore drainage and break the edema-inflammation-hypoxia loop. Mechanical-drug synergistic interventions—combining tension-reducing devices with pharmacotherapy—are rational given endothelial mechanosensitivity. Finally, integrating single-cell multi-omics with AI-based models may enable precise patient stratification. In summary, translating endothelial targeting into clinical benefit will require a shift from single-target, symptom-focused approaches to multi-dimensional, mechanism-based strategies addressing the full complexity of the keloid microenvironment.

## Glossary

ECM: Extracellular matrix
VECs: Vascular endothelial cells
LECs: Lymphatic endothelial cells
EndMT: Endothelial-to-mesenchymal transition
H&E: Hematoxylin and eosin
PlGF: Placental growth factor
EDs: Endothelial dysfunctions
HRE: Hypoxia response element
DAMPs: Damage-associated molecular patterns
IFN-γ: Interferon-γ
TNF: Tumor necrosis factor
ROS: Reactive oxygen species
VEGF-A: Vascular endothelial growth factor A
EGFR: Epidermal growth factor receptor
MA: Mechanically activated
MT1-MMP: Membrane-type matrix metalloproteinase-1
MMP-2: Matrix metalloproteinase-2
YAP: Yes-associated protein
TAZ: Transcriptional coactivator with PDZ-binding motif
NRP1: Neuropilin-1
NO: Nitric oxide
VEGF: Vascular endothelial growth factor
SP: Substance P
ET-1: Endothelin-1
AR: Androgen receptors
Ang II: Angiotensin II
AT1R: Angiotensin II type 1 receptor
AT2R: Angiotensin II type 2 receptor
ACE: Angiotensin-converting enzyme
TIMPs: Tissue inhibitors of metalloproteinases
eNOS: Endothelial nitric oxide synthase
TLR2: Toll-like receptor 2
EPCs: Endothelial progenitor cells
RAMP1: Receptor activity-modifying protein 1
5-FU: 5-Fluorouracil
HUVECs: Human umbilical vein endothelial cells
2ME2: 2-methoxyestradiol
BoNT-A: Botulinum toxin type A
ACEIs: Angiotensin-converting enzyme inhibitors
PDL: Pulsed dye laser
IPL: Intense pulsed light
S1P: sphingosine-1-phosphate
